# A Prospective, Randomized, Placebo-Controlled Study of a Combination of Simvastatin and Chemotherapy in Metastatic Breast Cancer

**DOI:** 10.1155/2020/4174395

**Published:** 2020-08-10

**Authors:** Hiba Alarfi, Lama A. Youssef, Maher Salamoon

**Affiliations:** ^1^Program of Clinical and Hospital Pharmacy, Department of Pharmaceutics and Pharmaceutical Technology, Faculty of Pharmacy, Damascus University, Damascus, Syria; ^2^Faculty of Pharmacy, International University for Science and Technology, Ghabagheb, Daraa, Syria; ^3^Al-Baironi Hospital, Ministry of Higher Education, Damascus, Syria

## Abstract

Preclinical studies support the anticancer activity of statins; however, the existing clinical evidence is inconsistent and not definitive. Our study aimed at evaluating a postulated cancer chemo-sensitizing effect of statin (simvastatin) in a cohort of metastatic breast cancer (MBC) patients. We designed a prospective, single-centered, randomized, double blinded, placebo-controlled trial that encompassed MBC patients with an ECOG Performance Status Scale ≤2 and scheduled to be treated with a chemotherapy regimen consisting of carboplatin and vinorelbine every 3 weeks at Al-Baironi Hospital, Damascus, Syria. Patients were enrolled between August 2011 and July 2012 and randomly allocated to receive a 15-day course of either simvastatin (40 mg) or placebo seven days prior to the first day of each chemotherapy cycle and then continued for eight days in each individual cycle. Primary endpoints were objective response rate (ORR) and toxicity, and the secondary endpoint was overall survival (OS). Eighty-two patients met the inclusion criteria and consented. ORR (35% vs. 32.5%) and predominant toxicity and grade ≥3 neutropenia (occurred in 30% vs. 40% of the patients) were not significantly different between simvastatin and placebo groups, respectively. Over a median follow-up of 44 months (range, 10–60), median OS was 15 months in the simvastatin group and 17 the in placebo group (hazard ratio (HR) = 1.16, 95% CI (0.70–1.91), *P*=0.57). Elevated baseline values of high-sensitivity C-reactive protein (hsCRP >10 mg/l), lactate dehydrogenase (LDH >480 U/L), and chemotherapy being ≥2^nd^ line were significantly associated with shorter OS for the total cohort in both Univariate and multivariate analyses. Our data prove a safe profile of simvastatin at 40 mg per day combined with carboplatin and vinorelbine in MBC patients but without any beneficial increase of tumor sensitivity to chemotherapy. Moreover, we demonstrated a strong clinical advantage of baseline values of hsCRP and LDH as useful prognostic tools in MBC patients. This trial is registered with ISRCTN12964275.

## 1. Introduction

Breast cancer is a major public health problem for women worldwide [1]. Early diagnosis and advances in treatments (i.e., chemotherapy, hormone therapy, and targeted therapies) have led to remarkable increases in survival rates. Nevertheless, a substantial number of patients will still develop metastases during the course of their disease [2]. Based on the currently available therapeutic options, cure of metastatic breast cancer (MBC) is a rather elusive goal, and palliative care can only help maintain quality of life while possibly prolonging survival [3]. Developing new therapeutic approaches for MBC is a time, patience, and diligence demanding research area [4]. Identifying new uses for established multimodes of action drugs (i.e., statins, metformin, and aspirin), also known as “drug repositioning”, represents a less costly and time sparing evolving approach [5].

Statins, or 3-hydroxy-3-methylglutaryl-Coenzyme A (HMG-CoA) reductase inhibitors, are well-established cholesterol-lowering agents commonly used in the primary and secondary prevention of cardiovascular disease [5]. Moreover, statins also inhibit the synthesis of essential isoprenoid intermediates required for activation of various intracellular signaling proteins known to play indispensable role(s) in multiple cellular processes, suggesting a pleiotropic nature of statins' effects. Beyond their lipid lowering properties, statins possess anti-inflammatory, antioxidant, and antiproliferative effects, which fed the growing interest in the therapeutic potential of statins in multiple treatment areas including oncology [6]. “Statin repositioning” was supported by *in vitro* and *in vivo* studies that have shown a wide range of anticancer activities, including induction of apoptosis, inhibition of tumor cell proliferation, and reduction of invasiveness and metastasis [6–8]. In addition, observational studies have provided substantial evidence that statin use is associated with a reduction in cancer incidence and mortality in several cancer types including breast cancer [9–11]. A few clinical studies have shown a positive role of lipophilic statins both as neoadjuvant therapy (i.e., before surgery) [12, 13] and in secondary prevention in breast cancer survivors [14]. Nevertheless, despite the growing evidence of synergistic effects of statins with chemotherapeutic drugs in other cancer types [15], no clinical studies investigated combination(s) of statins with standard treatment protocols in breast cancer.

Therefore, we designed this study to investigate the chemosensitizing effects of short-term treatment of simvastatin, at clinically relevant doses (40 mg), in MBC patients receiving a combination of carboplatin and vinorelbine. Although not universally accepted as the standard treatment for MBC [16], vinorelbine in combination with carboplatin is adapted in the clinical practice for MBC patients in “Al-Baironi” the major oncology Hospital in Syria. Since inflammation is a critical component of tumor progression and due to the well-established anti-inflammatory properties of statins [17], we sought to investigate the impact of statins on some inflammatory markers (high-sensitivity C-reactive protein (hsCRP) and lactate dehydrogenase (LDH)) and their prognostic potential in predicting therapeutic outcomes in MBC.

## 2. Materials and Methods

### 2.1. Trial Design and Eligibility

This was a prospective, single-centered, randomized, double blinded, placebo-controlled study. The study protocol was approved by the scientific research ethics committee at the Faculty of Pharmacy, Damascus University. The eligibility criteria were as follows: female patients attending the breast cancer unit at Al-Baironi Hospital, with confirmed diagnosis of metastases (stage IV) prior to commencing chemotherapy course consisting of carboplatin and vinorelbine; age between 20 and 75 years; adequate function of major organs (including cardiac, hepatic and renal functions); and an ECOG Performance Status score ≤2. Pregnant patients and those with previous treatment with statins or carboplatin and vinorelbine within 30 days of the study entry were excluded. All patients provided written informed consent and enrolled between August 2011 and July 2012. All treatments were double blinded to assure that neither oncologists were involved the study nor do participants know which type of preparation is administered. The follow-up lasted until death or the cutoff date of July 2017. Primary endpoints were objective response rate (ORR) and toxicity, and the secondary endpoint was overall survival (OS) over the follow-up period.

### 2.2. Treatment Protocol

Statin or placebo was provided in outpatient setting. Patients were assigned (1 : 1 or 2 : 2 ratio) to each treatment group ((carboplatin and vinorelbine) plus simvastatin or placebo) using randomization with metastasis sites as stratification factors. Simvastatin (40 mg) and placebo (provided in containers of identical shape and sequentially numbered) were generous gifts from ALFARES Pharmaceuticals Co. (Damascus, Syria). Chemotherapy regimen was conducted every 3 weeks according to the hospital protocol as follows: carboplatin (carboplatin “Ebewe”), area under the curve (AUC) 4, intravenously on day 1, and vinorelbine (Navelbine®) intravenously (25 mg/m^2^) or orally (60 mg/m^2^) on days 1 and 8 of each cycle. Simvastatin (40 mg) or placebo was administered orally once daily for 15 days, starting seven days prior to the first day of each chemotherapy cycle and continued to the eighth day to ensure that both chemotherapy and simvastatin have eight days of overlapping (i.e., as a combination therapy) and then followed by one-week rest.

Additionally, patients with bone metastases were treated with zoledronic acid via intravenous infusion (4 mg every 4 weeks), and palliative radiotherapy was allowed for brain metastases if needed. Study treatment was supposed to be continued in the absence of progression or until another termination criterion was met, including unacceptable toxicity, consent withdrawal, loss to follow-up, or death.

### 2.3. Response and Toxicity Assessment

Each patient underwent an initial evaluation within one week prior to commencing treatment. Evaluation encompassed full medical history, physical examination, chest X-ray, computed tomography (CT) scan, bone scan, magnetic resonance image (MRI) scan, and laboratory analyses, including complete blood counts (CBCs), creatine kinase (CK), creatinine, alanine aminotransferase (ALT), total cholesterol (TC), low-density lipoprotein cholesterol (LDL-C), high-density lipoprotein cholesterol (HDL-C), hsCRP, LDH and tumor markers; carcinoembryonic antigen (CEA), and cancer antigen 15-3 (CA15-3). With the exception of tumor markers, laboratory tests were performed at each cycle before chemotherapy administration on day 1, whereas TC, LDL-C, and HDL-C were repeated on day 8. To assess tumor progression, physical examination, tumor markers, and radiological studies were conducted at baseline and every three cycles, and bone scan was repeated by the end of the sixth cycle. Patients' response was classified according to the response evaluation criteria in solid tumor (RECIST) (version 1.1) as follows: complete response (CR), complete disappearance of clinical evidence of disease for a minimum of 8 weeks; partial response (PR), decrease in tumor burden ≥30%; stable disease (SD), decreased by <30% or increased by <20%; progressive disease (PD), increase in tumor burden by ≥20%; and nonevaluable response, due to specific reasons (e.g., early death or toxicity). ORR was calculated based on both CR + PR. Treatment related-toxicity was graded according to the Common Terminology Criteria for Adverse Events, version 4. In case of chemotherapy-related toxicity, including grade 2 neutropenia before the start of each cycle, treatment was delayed for 1 week. For grade 3/4 neutropenia, granulocyte colony-stimulating factor (G-CSF) was administered subcutaneously for 3 days at a dose of 5 *μ*g/kg. For simvastatin toxicity, treatment was planned to be discontinued if the serum transaminase was of more than 3 times the upper limit of the reference range or if the CK concentration was more than 5 times the upper limit of the reference range. OS was defined as time from study entry to death from any cause.

### 2.4. Statistical Analysis

We calculated the sample size of our study based on Simon's randomized Phase 2 design. According to Simon's method for calculating “Sample Size per Treatment for Binary Outcomes and 0.90 Correct Selection Probability,” the objective response rate for the chemotherapy line used in our study was previously reported by Iaffaioli (1995) to be approximately 41% (18); therefore, an increase of greater than 41 % would provide evidence of simvastatin effect. The intent was to enroll at least 74 patients in the two arms of the study in order to detect a 15 % absolute increase which would certainly indicate a superior response rate with 90% power and a two-sided type I error rate of 5%.

Statistical analysis was performed using Graphpad Prism® (version 5) except for Cox proportional hazard regression models that were performed using SPSS® (version 22) to calculate hazard ratios (HRs) and 95% confidence intervals (CIs) for both univariate and multivariate analyses. Between-group comparisons were performed using the chi-squared test for categorical variables, unpaired T test for normally distributed data, and Mann–Whitney test for data that were not normally distributed (hsCRP and LDH). Within each group, comparisons for lipid values (pre-vs. postchemotherapy) were performed using paired *T* test. Median overall survival was estimated using Kaplan–Meier analysis. Statistical significance was tested using the log-rank test, and two-tailed *P* value <0.05 was considered significant. The median follow-up was estimated using reverse censoring for overall survival.

## 3. Results

### 3.1. Patient Characteristics

The eighty-two MBC patients who enrolled were randomly assigned to treatment groups: 41 patients to the carboplatin and vinorelbine plus simvastatin and 41 patients to the carboplatin and vinorelbine plus placebo. The two treatment groups were well balanced in terms of their baseline characteristics as shown in Table 1.

The median age of all patients was 47.5 years (range 24–74 years). The ECOG-performance status was 1 in the majority of patients (73.17%). Fifty-six patients (68.29%) were positive for HER2 (human epidermal growth factor receptor-2), and 39 patients (47.56%) were ER/PR negative (estrogen receptor/progesterone receptor). Forty-two patients (51.22%) had two or more sites of metastases. The chemotherapy regimen was the first line in 37 patients (45.12%) and the second in 36 (43.9%).

### 3.2. Treatment Outcomes

**Figure 1:**
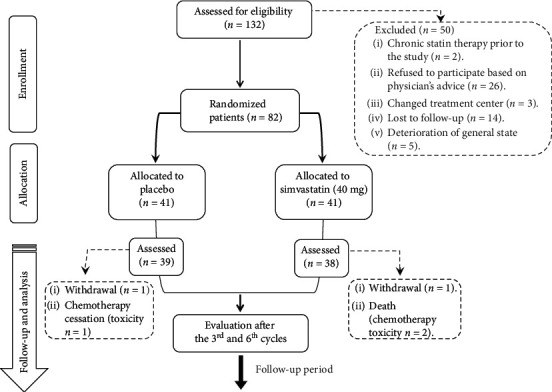
CONSORT flow chart.

Of the 82 patients enrolled in our study, 80 patients (97.6%) received at least 1 cycle of chemotherapy (median 4.5 cycles; range, 1–12 cycles). The remaining 2 patients (2.4%) withdrew consent. Only 77 patients were assessable for response by the end of the chemotherapy course, as 3 patients were classified not evaluable for response due to early death (*n* = 2) and chemotherapy toxicity (*n* = 1), as illustrated in Figure 1.

In the simvastatin group, one patient had a complete response, 13 patients had a partial response, and the ORR was 35%. Similarly, four patients had a complete response, nine patients had a partial response, and the ORR was 32.5% in the placebo group. No significant differences were found between the two treatment groups with regard to the response assessment results (*P*=0.57) (Table 2).

All patients who received at least one dose of therapy were assessable for toxicity. Most common grade 3 or higher adverse events were neutropenia (30% and 40% in the simvastatin and placebo group, respectively) and anemia (20% in both groups). The addition of simvastatin did not result in clinically significant increase in chemotherapy-related toxicities (no cases of CK elevated ≥ five times the upper limit of the normal range or ALT ≥ three times the upper limit of the normal range), as shown in Table 3.

**Figure 2:**
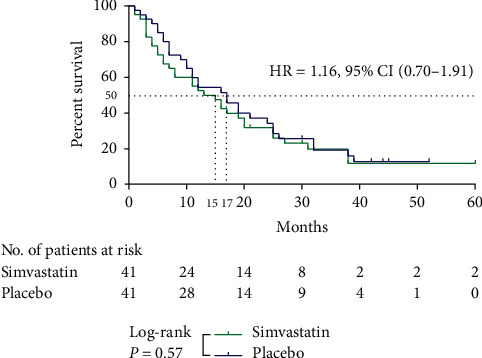
Survival for 60 months. The survival curve with reference to treatment groups (simvastatin vs. placebo) for MBC patients who were treated with palliative chemotherapy (carboplatin and vinorelbine). Median survival was estimated during a 60-month follow-up period using the Kaplan–Meier method. Statistical significance was assessed using the log-rank test, and two-tailed *P* value of <0.05 was considered significant.

By the end of a median follow-up period of 44 months (range, 10–60), an overall 65 fatalities were recorded. Median OS was not significantly different between the simvastatin group (15 months) and the placebo group (17 months) (HR = 1.16, 95% CI (0.70–1.91), *P*=0.57), as depicted in Figure 2.

We analyzed the difference(s) of overall survival outcomes between subgroups classified according to baseline characteristics including age, ECOG-PS, hormone receptors status, HER2 status, number of metastatic sites, chemotherapy line's grade, and baseline levels of CEA, CA15-3, hsCRP, and LDH. Univariate Cox models of survival revealed significantly shorter survival of MBC patients when they were <50 years old (*P*=0.026), having two metastatic sites or more (*P*=0.02), elevated baseline levels of hsCRP (*P*=0.002), LDH (*P*=0.002),CEA (*P*=0.016), or chemotherapy being ≥2^nd^ line (*P*=0.04). CA15-3 levels, ECOG-PS, HER2, and hormone receptors status did not significantly the impact survival (*P* > 0.05). After adjusting for other factors, multivariate Cox models proved that only elevated baseline levels of hsCRP (HR = 2.168, 95% CI (1.299–3.616), *P*=0.003) or LDH (HR = 2.213, 95% CI (1.273–3.845), *P*=0.005) and chemotherapy being ≥2^nd^ line (HR = 1.766, 95% CI (1.067–2.923), *P*=0.027) were still significantly predictive for shorter survival.  Table 4 demonstrates detailed results for univariate and multivariate analyses.

### 3.3. Impact of Simvastatin on Lipids and Inflammatory Markers

Neither TC nor LDL-C levels were significantly different at baseline between the two groups. However, simvastatin clearly induced a substantial drop in TC and LDL-C levels throughout the chemotherapy cycles in comparison with placebo (*P* ≪ 0.05) (see Table 1 in the Supplementary Material). Thorough follow-up of serum TC, LDL-C and HDL-C levels and pre- and postchemotherapy administration at each cycle revealed a significant decrease in TC and LDL-C levels at day 8 of each chemotherapy cycle, except for the sixth, in both simvastatin and placebo groups (summarized in Table 5). Noteworthy, following the one-week drug break, all patients recovered TC and LDL-C levels to within at least 77% of their baseline by the start of the next cycle, and this pattern persisted over all treatment cycles. Serum HDL-C levels did not differ significantly between the two groups at any cycle after simvastatin exposure.

No significant changes were observed in the levels of inflammatory markers (hsCRP and LDH) during the study in any of the treatment groups (see Table 2 in the Supplementary Material).

## 4. Discussion

Recently, the cholesterol lowering-independent or “pleiotropic” effects of statins have gained greater recognition, particularly in the area of cancer therapeutics [7]. Cumulative *in vivo* evidence and observational clinical studies suggest a therapeutic potential of statins in different cancer models [17]. Nevertheless, translating these findings into clinical studies faces multidimensional challenges comprising which statin to use, the timing (when or at what stage of cancer progression), the temporal frame (short-term versus long-term), and what cytotoxic agents/chemotherapy line, hormonal, or radiotherapy with which should a particular type of statin is coadministered. To our knowledge, this study represents the first clinical trial to utilize short-term simvastatin at therapeutically relevant dose (40 mg) in combination with carboplatin and vinorelbine in metastatic breast cancer patients.

The combination of carboplatin and vinorelbine is a one of the palliative treatments for MBC in Al-Baironi Hospital. Our study confirms the effectiveness of carboplatin plus vinorelbine with an ORR of 33.75%, a median OS of 16 months, and 35% grade ≥3 neutropenia for all patients. Iaffaioli et al. reported their experience with a same therapy regimen, the ORR was 41%, median OS was 16 months, and the principal toxicity was myelotoxicity and grade 3/4 leukopenia in 46% of advanced breast cancer patients [18].

Expectedly, the lipid lowering effect of simvastatin was evident by a significant decrease in total cholesterol and LDL-C levels compared with that of placebo. These findings suggest a good compliance with the study intervention and confirm the effectiveness of simvastatin in lowering lipids within a short-term (15 days) exposure frame.

Surprisingly, a significant drop in total cholesterol (range −3.26% to −9.43%, *P* ≪ 0.05) and LDL-C (range −2.82% to −12.73%, *P* ≪ 0.05) levels was observed between the first and eighth days of each of the treatment cycles in patients who received chemotherapy plus placebo. These observed simvastatin-independent changes in cholesterol levels are consistent with *in vitro* experiments, where that in acute myeloid leukemia (AML) samples exhibited abnormally increased demands for cholesterol following the exposure to cytotoxic agents (daunorubicin or cytarabine), a phenomenon described as “defensive adaptation” of cancerous cells to increase chemoresistance [19].

Within the 15-day simvastatin exposure in each cycle, no changes in hsCRP level was observed, suggesting that a dose of 40 mg of simvastatin for this short-term duration may not have been sufficient to exhibit anti-inflammatory benefit in MBC patients. These findings contradict others in coronary artery disease (CAD) patients, where short-term exposure of simvastatin lowered hsCRP [20]. We should note here that the hsCRP baseline levels are rather different between the two patient populations, (median [lower-upper quartile]; 8.18 [3.93–22.11] in MBC vs. 2.8 [1.3–4.8] mg/l or 1.1 [0.8–2.5] in female and male CAD patients, respectively). A significant reduction in serum CRP in breast cancer patients may demand a higher dose and/or longer duration of statin therapy (e.g. at least 3 months) to demonstrate a similar effect to that observed in CAD patients [21].

Despite the promising *in vitro* and *in vivo* evidence that provided support to antitumor effect(s) of statins in a variety of human malignancies [15], our findings prove that clinically relevant dose of simvastatin, added to carboplatin and vinorelbine course, has no clinical benefit in terms of outcome (i.e., ORR and median OS) in MBC patients. However, treatment with simvastatin in MBC proved to be very well tolerated, as no significant chemotherapy toxicity or simvastatin adverse effects were recorded. These findings are in agreement with a study by kim and colleagues that investigated a combination of statins with capecitabine and cisplatin in advanced gastric cancer, as no increase in progression free survival was reported [22]. Similarly, gemcitabine-simvastatin at 40 mg daily vs. gemcitabine-placebo resulted in no significant difference in time to progression in advanced pancreatic cancer patients [23]. The overall null results in our study and others may stem from statins' conflicting properties; on the one hand, they have antiproliferative effects, but on the other hand, they exhibit immune tolerance-promoting properties during tumor development. Therefore, statins might be concurrently inhibiting and promoting tumor growth [24]. Another explanation may arise from the pulsatile administration of statin (15 days every cycle), which may not be enough in terms of cholesterol deprivation of cancerous cells.

We decided the dose-level of simvastatin (40 mg) in combination with the carboplatin and vinorelbine course based on an observation that low concentrations of statins were capable of inducing apoptosis of microvascular endothelial cells and lowering VEGF serum levels, implicating a possible antiangiogenic role for statin in cancer treatment [22, 25]. Of interest, statins were proved to induce apoptosis through activation of the JNK-signaling pathway, and since inhibition of JNK activation is a major mechanism beyond tumor resistance to platinums and Vinca alkaloid, the addition of statins to either of these drugs is speculated to help overcome chemoresistance [26, 27].

There have been only a few reports on prognostic factors in patients with metastatic disease [28]. Notably, not all studies agreed on the same set of risk factors explaining variation in prognosis following breast cancer metastasis [29]. Some studies have shown that age, number of metastatic sites, ER/PR and HER2 status, ECOG-PS, and baseline values of CEA and CA15-3 are valuable prognostic factors [28–31]. Nevertheless, our study did not provide support for a significant influence of any of these factors on survival in multivariate analysis. Variations in prognostic factors in terms of the patients' selection, presence of clinical covariates, rate of patients' lost to follow-up, lines of chemotherapy, and statistical method for analysis [29, 32] may explain the differences between our results and those from previous ones.

Nevertheless, our findings prove that increased baseline serum concentrations of hsCRP may serve as a predictor for poor prognosis among MBC patients. This result is consistent with that of Albuquerque et al.'s study [33], Murri et al.'s study [34], and Petekkaya et al.'s study [35].

On the other hand, Swenerton et al. were the first to report the clinical importance of serum lactate dehydrogenase in predicting survival of MBC patients [32]. Our results reinforced that elevated serum LDH levels significantly correlate with poorer survival among MBC patients [35,36].

No global consensus exists regarding the ideal treatment strategy for MBC. Thus, once the first line failed, second and later lines are adapted, reflecting the clinically challenging picture of progressive disease [2]. Our data showed that the grade of chemotherapy line had significant impact on survival of MBC patients.

## 5. Conclusions

The present study is the first, to the best of our knowledge, to investigate the proposed chemosensitizing effect of simvastatin added on to carboplatin and vinorelbine in metastatic breast cancer. Adding simvastatin to this combination did not seem to provide any additional clinical benefit and also did not result in any significant increase in toxicity. We were able to show that inflammatory markers, such as CRP and LDH, can be used to predict prognosis in patients with metastatic breast cancer. We recognize our study's limitations concerning the generalization of the conclusions based on data originated from a small-sized sample of MBC patients receiving one line of chemotherapy. Larger population-based studies on different chemotherapy agents are needed to confirm or refute any prognostic significance in MBC patients.

## Figures and Tables

**Table 1:** Baseline patients' characteristics.

Characteristics		Chemotherapy + simvastatin (*n* = 41)	Chemotherapy + placebo (*n* = 41)	*P* value	Total (*n* = 82)
		*N* (%)			*n* (%)
Age (years)					
Median		47	49	0.73	47.5
Range		28–74	24–71		24–74

BMI (kg/m^2^)					
<18.5		1 (2.44)	0 (0)	0.46	1 (1.21)
18.5–24.9		9 (21.95)	11 (26.83)		20 (24.39)
25–29.9		15 (36.59)	19 (46.34)		34 (41.46)
≥30		16 (39.02)	11 (26.83)		27 (32.92)

No. of metastatic sites					
1		23 (56.10)	17 (41.46)	0.23	40 (48.78)
2		17 (41.46)	20 (48.78)		37 (45.12)
3		1 (2.44)	4 (9.75)		5 (6.09)

Site of metastases					
Bone		5 (12.20)	6 (14.63)	0.54	11 (13.41)
Liver		8 (19.51)	3 (7.32)		11 (13.41)
Lung		4 (9.76)	3 (7.32)		7 (8.54)
Brain		0 (0)	1 (2.44)		1 (1.22)
Skin/chest wall		2 (4.88)	2 (4.88)		4 (4.88)
Lymph node		4 (9.76)	2 (4.88)		6 (7.32)
Multiple sites		18 (43.9)	24 (58.54)		42 (51.22)

ECOG-PS					
0		4 (9.75)	4 (9.75)	0.84	8 (9.75)
1		29 (70.73)	31 (75.61)		60 (73.17)
2		8 (19.50)	6 (14.63)		14 (17.07)

Hormone receptor					
ER+ PR+		14 (34.15)	13 (31.71)	0.46	27 (32.92)
ER− PR−		18 (43.90)	21 (51.22)		39 (47.56)
ER+ PR−		2 (4.88)	4 (9.76)		6 (7.32)
ER− PR+		5 (12.20)	1 (2.44)		6 (7.32)
Unknown		2 (4.88)	2 (4.88)		4 (4.88)

HER 2					
HER+		30 (73.17)	26 (63.41)	0.6	56 (68.29)
HER−		9 (21.95)	13 (31.71)		22 (26.82)
Unknown		2 (4.88)	2 (4.88)		4 (4.88)

Chemotherapy line					
1^st^ line		18 (43.90)	19 (46.34)	0.37	37 (45.12)
2^nd^ line		17 (41.46)	19 (46.34)		36 (43.90)
≥3^rd^ line		6 (14.63)	3 (7.32)		9 (10.97)

**Table 2:** Efficacy outcomes.

Best response	Chemotherapy + simvastatin (*n* = 40)	Chemotherapy + placebo (*n* = 40)	*P* value
	*n* (%)		
Complete response	1 (2.5)	4 (10)	0.57
Partial response	13 (32.5)	9 (22.5)	
Stable disease	10 (25)	11 (27.5)	
Progressive disease	14 (35)	15 (37.5)	
Not evaluable	2 (5)	1 (2.5)	

The assessment did not include the withdrawn consent patients (*n* = 2)

**Table 3:** Most common grades 1 to 4 adverse events of chemotherapy and adverse events of special interest to simvastatin.

Adverse events *n* (%)	Chemotherapy + simvastatin (*n* = 40)		Chemotherapy + placebo (*n* = 40)	
	Grade 1/2	Grade 3/4	Grade 1/2	Grade 3/4
Anemia	34 (85)	8 (20)	32 (80)	8 (20)
Thrombopenia	5 (12.5)	1 (2.5)	4 (10)	2 (5)
Neutropenia	22 (55)	12 (30)	18 (45)	16 (40)
Clinical hemorrhage	—	—	1 (2.5)	—
Injection site reaction	7 (17.5)	—	5 (12.5)	—
Rash	—	—	1 (2.5)	—
Left ventricle function	1 (2.5)	—	—	1 (2.5)
Stomatitis	—	—	1 (2.5)	—
Creatinine elevation	6 (15)	—	5 (12.5)	—
Simvastatin special adverse events		—		
ALT elevation^*∗*^	12 (30)	—	13 (32.5)	—
CK elevation^#^	1 (2.5)	—	4 (10)	—

^*∗*^None of the elevated levels of ALT exceed more than 3 times the upper limit of the reference range. ^#^None of the elevated levels of CK exceed more than 5 times the upper limit of the reference ranges *P* value >>0.05 for all comparisons

**Table 4:** Factors associated with overall survival for total cohort.

Variables	Univariate analysis		Multivariate analysis	
	Hazard ratio (95% CI)	*P* value	Hazard ratio (95% CI)	*P* value
hsCRP (mg/l)				
>10 vs. ≤ 10	2.210 (1.339–3.647)	0.002	2.168 (1.299–3.616)	0.003

LDH (U/l)				
>480 vs. ≤ 480	2.335 (1.351–4.036)	0.002	2.213 (1.273–3.845)	0.005

CEA (ng/ml)				
>5 vs. ≤ 5	1.842 (1.119–3.033)	0.016		

CA15-3 (U/ml)				
>30 vs. ≤ 30	1.289 (0.673–2.469)	0.444		

Age (years)				
≥50 vs. <50	0.555 (0.330–0.932)	0.026		

ECOG-PS				
1/2 vs. 0	2.352 (0.939–5.893)	0.068		

HER2				
(−) vs. (+)	1.009 (0.583–1.747)	0.973		

Hormone receptors (HRs)				
(−) vs. (+)	0.681 (0.412–1.124)	0.133		

No. of metastatic sites				
≥2 sites vs. 1 site	1.802 (1.095–2.965)	0.020		

Chemotherapy line				
≥2^nd^ line vs. 1^st^ line	1.692 (1.025–2.795)	0.040	1.766 (1.067–2.923)	0.027

**Table 5:** Differences in TC, LDL-C, and HDL-C levels during each chemotherapy cycle.

Variable	Simvastatin		Placebo	
	Differences between 1^st^ and 8^th^ day of each chemotherapy cycle			
	Mean (%)	*P* value	Mean (%)	*P* value
TC (mg/dl)				
Cycle 1	−15.67 (−9.54%)	0.007	−18.13 (−8.57%)	0.003
Cycle 2	−15.91 (−9.07%)	0.013	−20.47 (−9.43%)	0.0002
Cycle 3	−13.58 (−7.81%)	0.018	−13.52 (−6.6%)	0.017
Cycle 4	−16.05 (−9.01%)	0.013	−19.45 (−8.71%)	0.002
Cycle 5	−14.29 (−8.43%)	0.016	−20.13 (−9.16%)	0.001
Cycle 6	−8.17 (−4.85%)	0.3	−7 (−3.26%)	0.3

LDL-C (mg/dl)				
Cycle 1	−10.42 (−11.75%)	0.006	−16 (−12.73%)	0.001
Cycle 2	−16.85 (−16.77%)	0.0006	−8.71 (−6.7%)	0.014
Cycle 3	−10.02 (−9.84%)	0.018	−9.84 (−7.85%)	0.006
Cycle 4	−11.41 (−10.94%)	0.008	−15.59 (−11.4%)	0.0002
Cycle 5	−6.29 (−6.44%)	0.2	−7.31 (−5.53%)	0.056
Cycle 6	−7.17 (−7.31%)	0.29	−3.85 (−2.82%)	0.6

HDL-C (mg/dl)				
Cycle 1	0.52 (1.06%)	0.5	−3.47 (−6.77%)	0.1
Cycle 2	0.06 (0.12%)	0.9	−4.65 (−8.58%)	0.06
Cycle 3	0.03 (0.07%)	0.9	−0.55 (−1.12%)	0.8
Cycle 4	0.22 (0.46%)	0.9	0.23 (0.45%)	0.9
Cycle 5	−0.21 (−0.45%)	0.9	−5.31 (−9.38%)	0.03
Cycle 6	0.23 (0.48%)	0.9	−0.46 (−0.84%)	0.8

## Data Availability

The datasets used and/or analyzed during the current study are available from the corresponding author on reasonable request.

## Abbreviations

ALT: Alanine aminotransferase
AML: Acute myeloid leukemia
AUC: Area under the curve
CA15-3: Cancer antigen 15–3
CAD: Coronary artery disease
CBC: Complete blood counts
CEA: Carcinoembryonic antigen
CIs: Confidence intervals
CK: Creatine kinase
CR: Complete response
CT: Computed tomography
ER/PR: Estrogen receptor/progesterone receptor
G-CSF: Granulocyte colony-stimulating factor
HDL-C: High-density lipoprotein cholesterol
HER2: Human epidermal growth factor receptor-2
HMG-CoA: 3-Hydroxy-3-methylglutaryl-Coenzyme A
HRs: Hazard ratios
hsCRP: High-sensitivity C-reactive protein
LDH: Lactate dehydrogenase
LDL-C: Low-density lipoprotein cholesterol
MBC: Metastatic breast cancer
MRI: Magnetic resonance image
ORR: Objective response rate
OS: Overall survival
PD: Progressive disease
PR: Partial response
RECIST: Response evaluation criteria in solid tumor;
SD: Stable disease
TC: Total cholesterol.

## Supplementary Materials

Supplementary Materials Table 1: serum levels of TC, LDL TC, LDL-C, and HDL-C were measured on day 1 and day 8 of each cycle, and comparisons were made between simvastatin and placebo groups (DOCX 15 kb). Table 2: serum levels of hsCRP and LDH throughout and LDH were measured in each cycle, and comparisons were made between simvastatin and placebo groups (DOCX 13 kb).
