# Supplementary material for: A Prospective, Randomized, Placebo-Controlled Study of a Combination of Simvastatin and Chemotherapy in Metastatic Breast Cancer
Source: J Oncol. 2020 Aug 10;2020:4174395. doi: 10.1155/2020/4174395 (PMC7436279; doi:10.1155/2020/4174395)
Supplement: Supplementary Materials — Table 1: serum levels of TC, LDL TC, LDL-C, and HDL-C were measured on day 1 and day 8 of each cycle, and comparisons were made between simvastatin and placebo groups (DOCX 15 kb). Table 2: serum levels of hsCRP and LDH throughout and LDH were measured in each cycle, and comparisons were made between simvastatin and placebo groups (DOCX 13 kb). [file 4174395.f1.pdf]

Table 1. Serum levels of TC, LDL-C and HDL-C throughout the study cycles

Variable	Simvastatin	Placebo		Simvastatin	Placebo	
	1 st day chemotherapy			8 th day chemotherapy		
	Mean ± SD		*P* value	Mean ± SD		*P* value
**TC (mg/dl)**						
Baseline (day -7)	214.5± 49.1	204.1± 45.6	0.39			
Cycle 1	164.3± 39.7	211.6± 32.3	**0.0003**	153.6± 34.3	185± 35.1	**0.004**
Cycle 2	175.3± 42.6	217.1± 50.3	**0.0003**	155.5± 37.2	197.4± 47.1	**0.0002**
Cycle 3	173.8± 37.9	204.7± 45.7	**0.003**	162.4± 29.5	192.5± 36.3	**0.002**
Cycle 4	178.1± 34.5	223.3± 47.9	**0.0002**	163.5± 28.5	205.6± 44.8	**0.0006**
Cycle 5	169.6± 37.9	219.7± 44.9	**0.001**	158.9± 19.9	201.7± 45.4	**0.003**
Cycle 6	168.6± 40.1	214.9± 48.3	**0.008**	154± 27.9	211.3± 48.9	**0.002**
**LDL-C (mg/dl)**						
Baseline (day -7)	127.3± 38.4	124.5± 35.1	0.77			
Cycle 1	88.71± 28	125.7± 24.9	**0.0001**	84.12± 31.2	107.2± 23.5	**0.008**
Cycle 2	100.5± 30.9	129.7± 33.2	**0.0003**	81.3± 27.7	119.9± 36.3	**< 0.0001**
Cycle 3	101.8± 28.9	125.4± 34.5	**0.003**	91.6± 22.4	116.6± 31.3	**0.002**
Cycle 4	104.3± 27.3	136.8± 36.4	**0.0004**	93.2± 17	122.9± 36.1	**0.001**
Cycle 5	97.6± 23.4	132.1± 35.3	**0.002**	93.1± 19.3	125± 38.8	**0.009**
Cycle 6	98.1± 18.4	136.5± 39.6	**0.002**	89.1± 17.6	138.9± 38.2	**0.0004**
**HDL-C (mg/dl)**						
Baseline (day -7)	51.03± 13.3	51.3± 11.5	0.93			
Cycle 1	49.2± 13.7	51.2± 11.6	0.63	49.97± 11.5	46.2± 14	0.31
Cycle 2	48.3± 9.9	54.2± 14.6	**0.04**	47.2± 11.3	49.7± 12.6	0.40
Cycle 3	43.5± 9.2	48.97± 14.3	0.06	44.4± 7.6	48.4± 10.2	0.11
Cycle 4	48.3± 10.8	51.2± 9.8	0.3	48.4± 10.6	51.7± 12.3	0.33
Cycle 5	46.4± 10.6	56.6± 15.2	0.02	46.6± 8.9	53.4± 15.5	0.16
Cycle 6	48.3± 17.1	54.7± 16.3	0.29	47.3± 16.6	53.5± 16.3	0.35

Table 2. Serum levels of hsCRP and LDH throughout the study cycles

Variable	Chemotherapy + simvastatin	Chemotherapy + placebo	*P* value
**hsCRP (mg/L)**	**Median (minimum- maximum)**		
Baseline (day -7)	7.98 (0.51-125.6)	8.93 (0.49-127.1)	0.54
Cycle 1	7.5 (0.579-86.19)	8.51 (0.5-99.29)	0.87
Cycle 2	10.41 (1.2-65.42)	8.215 (1.06-37.9)	0.26
Cycle 3	11.95 (1.02-129)	8.715 (0.31-43.28)	0.25
Cycle 4	10.8 (0.09-166.9)	9.76 (0.76-57.4)	0.23
Cycle 5	13.47 (2.46-183.7)	4.7 (1.1-34.35)	0.11
Cycle 6	13.98 (1-152.4)	5.1 (1.07-18.54)	0.13
**LDH (U/L)**			
Baseline (day -7)	589.5 (219-1830)	538 (26-1920)	0.42
Cycle 1	532 (276-1880)	597.5 (341-1905)	0.60
Cycle 2	489 (133-1099)	530 (234-1990)	0.59
Cycle 3	533.5 (151-2011)	518.5 (6.74-2892)	0.94
Cycle 4	462 (179-2007)	500 (325-2869)	0.76
Cycle 5	484 (282-1457)	489 (376-1811)	0.62
Cycle 6	481.5 (113-1131)	437 (319-1125)	0.58
